# Mapping of the *Cladosporium fulvum* resistance gene *Cf-16*, a major gene involved in leaf mold disease in tomato

**DOI:** 10.3389/fgene.2023.1219898

**Published:** 2023-07-27

**Authors:** Dongye Zhang, Huijia Li, Guan Liu, Libo Xie, Guojun Feng, Xiangyang Xu

**Affiliations:** ^1^ College of Advanced Agriculture and Ecological Environment, Heilongjiang University, Harbin, China; ^2^ Laboratory of Genetic Breeding in Tomato, College of Horticulture and Landscape Architecture, Northeast Agricultural University, Harbin, China; ^3^ Horticultural Sub-Academy, Heilongjiang Academy of Agricultural Sciences, Harbin, China

**Keywords:** tomato, mapping, *Cladosporium fulvum*, resistance gene Cf-16, BSA, SSR markers

## Abstract

Tomato (*Solanum lycopersicum*) is widely cultivated and consumed worldwide. Tomato leaf mold, caused by *Cladosporium fulvum*, is one of the most devastating diseases in tomato production. At present, some tomato leaf mold resistance (*Cf* series) genes used in production gradually lose resistance due to the continuous and rapid differentiation of *C. fulvum* physiological races. The *Cf-16* gene derived from the “Ontario7816” tomato cultivar has shown effective resistance in field trials for many years, but few studies have reported on the mapping of the *Cf-16* gene, which has not been cloned, limiting its utilization in tomato breeding. Here, we mapped *Cf-16* using a novel comprehensive strategy including bulk segregation analysis (BSA), genome resequencing and SSR molecular markers. A genetic analysis revealed that *Cf-16* resistance in “Ontario7816” is controlled by one major dominant locus. The *Cf-16* gene was mapped in a region of 2.6 cM at chromosome 6 between two markers, namely, TGS447 and TES312, by using an F_2_ population from a cross between the resistant cultivar “Ontario7816” and susceptible line “Moneymaker.” Two nucleotide-binding-site-leucine-rich repeat (NBS-LRR) resistance genes, namely, XM_004240667.3 and XM_010323727.1, were identified in this interval. They are strong candidates for the *Cf-16* gene. The mapping of *Cf-16* may speed up its utilization for breeding resistant tomato varieties and represents an important step forward in our understanding of the mechanism underlying resistance to tomato leaf mold.

## Introduction

Tomato (*Solanum lycopersicum* L.) is a global vegetable cultivated and consumed worldside that has outstanding nutritional. Because of its rich flavor, it is also often used as a fruit (Kim et al., 2017). Leaf mold disease caused by *Cladosporium fulvum* is considered to be one of the most devastating diseases in tomato crops and has threatened tomato-growing areas worldwide, especially for tomato grown in protected areas. This pathogen reduces both fruit yield and quality and sometimes even kills tomato plants (Thomma et al., 2005; Mesarich et al., 2014). In the early stages, tomato leaf mold disease symptoms appear as irregular or oval light yellow chlorotic spots on the backs of the leaves. As the disease progresses, the affected leaves grow gray‒brown or dark-brown velvety mold layers. When the disease is severe, the leaves senesce, wither and fall off prematurely (Zhao et al., 2022). However, it is increasingly difficult to control this disease in tomato-growing regions around the world. Thus, breeding for tomato leaf mold resistance will provide an effective and environmentally friendly alternative to chemical control. *C. fulvum* is the causal organism of tomato leaf mold, a fungal disease first described by Cooke (1883). The differentiation rate of *C. fulvum* physiological races is very rapid, and new races are continuously isolated and identified (Enya et al., 2009; Iida et al., 2010; Li et al., 2015). Thus, the resistance effectiveness of some tomato leaf mold resistance (*Cf* series) genes used in breeding production has gradually been undermined. Therefore, the mining, cloning and application of new *Cf* genes have become particularly critical.

*Cf* genes are a class of functional genes with typical AVR recognition ability (Rivas and Thomas, 2005). At least 24 *Cf* genes, named *Cf-1* ∼ *Cf-24*, have been identified by Dr. Kerr’s laboratory. The susceptible cultivar ‘Moneymaker’ is known as a *Cf-0* cultivar what means that it lack resistance genes. These *Cf* genes are derived from tomato and its wild relatives (Kanwar et al., 1980). At present, some *Cf* genes have been cloned using methods such as transposon tagging and map-based cloning, including *Cf-2* (*Hcr2-2B*, *Hcr2-2C*), *Cf-5*, *Cf-4* (*Hcr9-4D*), *Cf-4E* (*Hcr9-9B*), *Cf-9* (*Hcr9-9C*), and *9DC* (*Hcr9-M205*) (Zhao et al., 2019). Some *Avr* genes (*Avr2*, *Avr4*, *Avr4E*, *Avr5*, *Avr9*, Ecp1, Ecp2, Ecp4, Ecp5, Ecp6, and Ecp7) corresponding to *Cf* genes have also been isolated and identified (Zhao et al., 2022). *Cf* genes encode transmembrane glycoproteins with typical extracellular leucine-rich repeats (LRRs). The different LRR numbers in different *Cf* genes determine the ability of different *Cf* genes to recognize different physiological races of the pathogen (Kruijt et al., 2005).

DNA markers have been widely used in identifying and mapping plant disease resistance genes. In recent years, single-nucleotide polymorphism (SNP) markers have been widely used to identify markers associated with important traits, including tomato disease resistance (Yang et al., 2017; Liu et al., 2019). Bulked segregant analysis (BSA) can rapidly detect specific genes or genomic regions. In particular, the combination of BSA and genome resequencing accelerates the cloning of genes responsible for important traits (Michelmore et al., 1991; Zou et al., 2016). Moreover, Kompetitive allele-specific PCR (KASP) markers converted from special SNPs are more flexible and efficient than the original SNP markers for use in genotyping and marker-assisted selection (MAS) (Liu et al., 2019; Yang et al., 2022). However, simple sequence repeat (SSR) markers are still used to study traits of interest by linkage analysis (Rui et al., 2017).

In this study, we explored the genetic characteristics of the *Cf-16* gene by developing a large F_2_ population and BC_1_ populations. Notably, this is the first time that BSA combined with genome resequencing technology and molecular markers has been used for mapping the tomato *Cladosporium fulvum* resistance gene *Cf-16*. This study will be valuable for *Cf-16* cloning and resistance breeding in tomato.

## Materials and methods

### Plant materials and *C. fulvum* inoculation

Resistant female “Ontario7816” (P_1_, kindly provided by the Institute of Vegetables and Flowers, Chinese Academy of Agricultural Science), whose genome contain the *Cf-16* gene, was crossed with the susceptible male “Moneymaker” (P_2_, kindly provided by the Tomato Genetic Resource Center, LA2706). The F_1_ individuals were then self-crossed to harvest F_2_ seeds, and the BC_1_ plants were generated by backcrossing the F_1_ plants with “Ontario7816” and “Moneymaker.” All plants were grown in the greenhouse under favorable conditions.

Ten *C. fulvum* physiological races (1.2, 1.2.3, 1.2.3.4, 1.2.4, 1.3.4, 2.3, 1.3, 1.4, 1.2.3.4.5, and 1.2.3.4.9) provided by the tomato research group of Horticulture College, Northeast Agricultural University, were used to test to characterize the resistance responses of “Ontario7816” and “Moneymaker”.

At the 5–6 leaf stage, all P_1_, P_2_, F_1_, F_2_, and BC_1_P_2_ plants were inoculated with *C. fulvum* race 1.2.3.4., which is a predominant physiological race in Heilongjiang Province, China. The plants were assessed for disease severity at 15 days postinoculation. Inoculation and assessment of disease severity ratings were performed as described by Xue et al. (2017). The plants were visually assessed for the severity of symptoms on a scale of 0–9 points. Plants with a disease index of 0–3 were regarded as resistant, and those with a score of 5–9 were regarded as susceptible.

### DNA extraction, resequencing and association analysis

After inoculation, the F_2_ plants were used for genetic analysis and bulked segregant analysis (BSA). The parents (P_1_ and P_2_) and the F_2_ lines were prepared for genome resequencing and molecular marker detection, respectively. The resistant pool (F_2_R-pool, 25 resistant plants) and the susceptible pool (F_2_S-pool, 25 susceptible plants) were built by screening resistant and susceptible plants from the 726 F_2_ individuals. The cetyl-trimethylammonium bromide (CTAB) method was used for DNA extraction from young leaves, including those of the parents and the F2 lines. Bulked DNA samples were also subjected to the CTAB method by mixing equal amounts of DNA at a final concentration of 200 mg (Allen et al., 2006).

The resequencing and associated analysis were performed by BGI Tech (Shenzhen, China). The samples were constructed to generate 200–300 bp small fragment libraries for upsequencing on BGISEQ. The raw data were filtered to obtain clean data by the BGI in-house filter SOAPnuke. Subsequently, the clean data were aligned to the *S. lycopersicum* genome sequence (ftp://ftp.ncbi.nlm.nih.gov/genomes/all/GCF/000/188/115/GCF_000188115.3_SL2.50) using Burrows-Wheeler Aligner (Li and Durbin, 2010). SNP and InDel detection processes were performed with Genome Analysis Toolkit (GATK) (Li et al., 2009), and we used an analysis tool developed by BGI to perform annotation and classification. The SNP-index method was used for the association analysis. The SNP index values of the resistant and susceptible pools were calculated using the resistant female “Ontario7816” as a reference.

### SSR molecular marker analysis

Based on the candidate region of the association analysis, a total of 58 SSR markers from the Sol Genomics Network (SGN, http://solgenomics.net/) database were screened (Supplementary Table S1). SSR molecular markers were performed using the parents, F_1_ and 303 F_2_ individuals. Genetic linkage mapping was performed by JoinMap4 software (Ooijen and Van, 2006). Based on the electrophoresis results of 8% non-denaturing polyacrylamide gels.

### General strategy used for mapping the *Cf-16* gene

The general strategy of mapping the *Cf-16* gene in “Ontario7816” is illustrated in Supplementary Figure S1. To determine the inheritance pattern of *Cf-16* resistance in “Ontario7816” first, 726 F_2_ individuals and 106 BC_1_P_2_ individuals were planted and inoculated with *C. fulvum* race 1.2.3.4. The segregation ratios of resistant and susceptible phenotypes were recorded and further examined by the chi-square test. Next, to explore the rough position of the *Cf-16* gene, bulked segregation analysis (BSA) combined with parental resequencing was performed. To further map the *Cf-16* gene, 303 F_2_ individuals were planted and further screened with 58 SSR markers from the SGN (Supplementary Figure S1 **)**.

## Results

### Resistance and genetic analysis of the *Cf-16* gene

To identify the resistance potential of the *Cf-16* gene, we tested the resistance response of “Ontario7816” and “Moneymaker” to ten different races of *C. fulvum* (Table 1). “Moneymaker” showed susceptibility to all these physiological races of *C. fulvum*, while “Ontario7816” demonstrated resistance to all these races. These findings indicate that “Ontario7816” has highly resistant to *C. fulvum* and is suitable for future breeding work for disease resistance.

**TABLE 1:** The resistance response of “Ontario7816” and “Moneymaker” to ten different physiological races of *C. fulvum*.

Physiological races	Moneymaker (*Cf-0*)		Ontario7816(*Cf-16*)	
	Disease index	Resistance level	Disease index	Resistance level
1.2	65.4	HS	24.2	R
1.2.3	50.1	S	17.6	R
1.2.3.4	55.5	HS	20.4	R
1.2.4	76.1	HS	18.5	R
1.3.4	58.4	HS	9.8	R
2.3	70.7	HS	34.5	R
1.3	59.9	HS	16.4	R
1.4	54.2	HS	19.9	R
1.2.3.4.5	57.3	HS	22.6	R
1.2.3.4.9	61.9	HS	18.9	R

R, resistant; S, susceptible; HS, highly susceptible.

**FIGURE 1:**
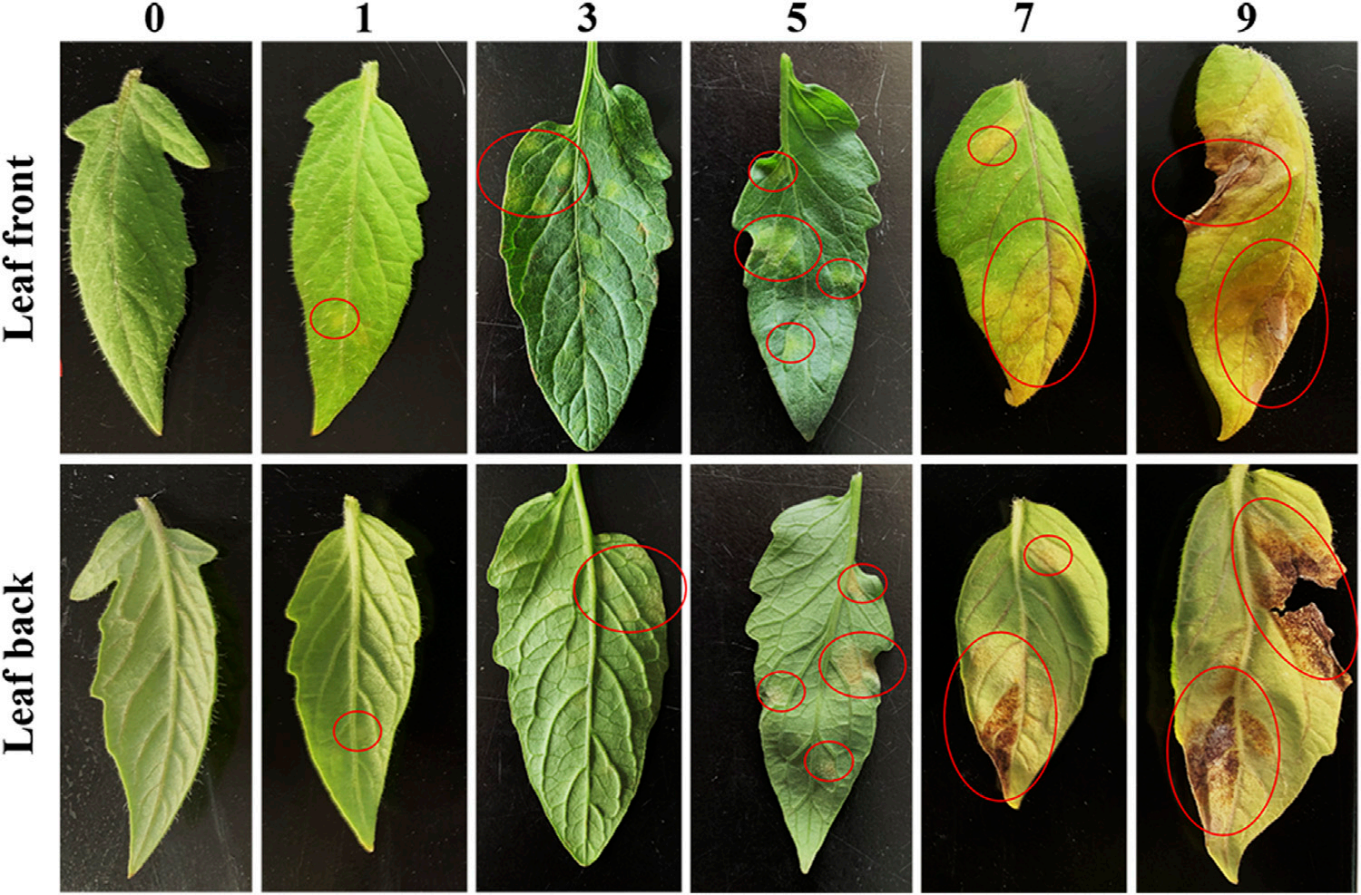
Symptom rating index (0–9) of tomato leaf mold.

Then, we performed a disease assay on the two parents, F_1_, F_2_ and BC_1_P_2_ individuals at 15 days postinoculation with the predominant physiological race 1.2.3.4 in Heilongjiang Province (Figure 1). “Ontario7816” and F_1_ plants were resistant to *C. fulvum* race 1.2.3.4, while “Moneymaker” plants were susceptible. Chi-square analysis showed that the segregation ratio of resistant and susceptible individuals of the F_2_ population was 3:1. The resistant and susceptible BC_1_P_2_ plants segregated according to the expected ratio of 1:1 (Table 2). This result confirms that the *Cf-16* gene confers resistance via a single dominant gene.

**TABLE 2:** Genetic analysis of *Cf-16* disease resistance in different generations.

Generation	No. of total plants	No. of resistant plants	No. of susceptible plants	The segregation ratio of R:S	χ2
P1 (Ontario7816)	46	46	0		
P2 (Moneymaker)	42	0	42		
F1	26	26	0		
F2	726	551	175	3.149:1	0.2645
BC1P2	106	55	51	1.078:1	0.0849

^χ2^_0.05, 1_ = 384.

### Parental genome sequencing and SNP-index association analysis

A total of 208.96 Gb clean data were obtained by Beijing Genomics Institute (BGI) sequencing, including 109.72 Gb from the parents and 99.26 Gb from the resistant and susceptible pools, all reads were of high quality (92.85% > Q30 > 90.45%) and with a stable GC content (36.28% > GC > 35.32%). The effective depths for the parents and the two F_2_ pools were between 56.23 and 67.16. The quality of the sequencing is high and the data are reliable and could be used for subsequent analysis (Table 3).

**TABLE 3:** Summary of the sequencing data.

Sample	GC_rate (%)	Q20_rate (%)	Q30_rate (%)	Bases (Gb)	Coverage_rate (%)	Map_reads_rate (%)	Effective_depth
P1	36.16	97.7	90.45	54.4	89.04	99.43	66.03
P2	35.32	97.89	91.02	55.32	89.29	99.83	67.16
R_F2	36.28	98.34	92.85	52.93	89.48	97.63	64.25
S_F2	35.38	98.01	91.71	46.32	89.47	99.75	56.23

Coverage_rate, the ratio of the sequence to the entire genome; Map_reads_rate, the ratio of the number of reads to the reference genome to the number of reads of clean data; Effective_depth, the ratio of the number of bases aligned to the reference genome to the size of the effective genome (N is not included in the reference sequence).

**FIGURE 2:**
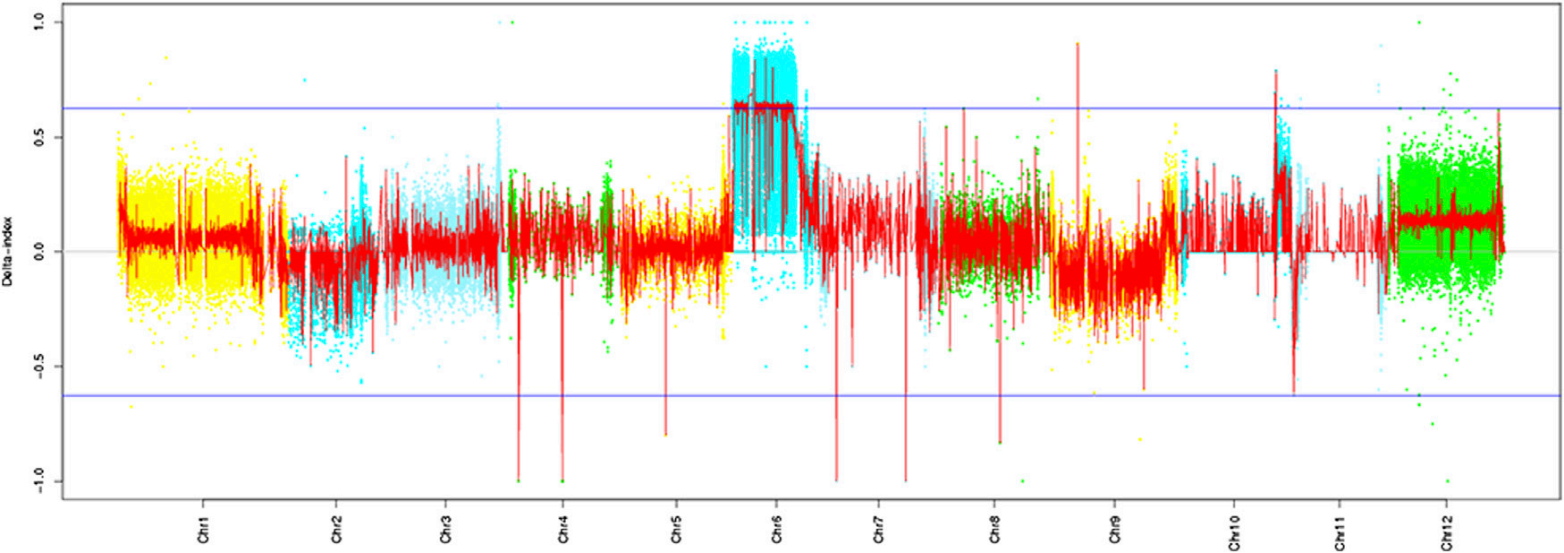
SNP-index algorithm to map the *Cf-16* gene.

Based on the resequencing results, we calculated the SNP index values of the two mixed pools using the resistant parent “Ontario7816” as the reference. The *Cf-16* gene was localized on tomato chromosome 6 by ΔSNP index analysis (Figure 2). Further analysis revealed an SNP imbalance between 11 and 38 Mb on chromosome 6, while the ΔSNP index of this region was greater than the threshold value at the 99% confidence level. Therefore, 11–38 Mb of chromosome 6 is the preliminary localization interval of the *Cf-16* gene for leaf mold resistance in tomato.

### Further mapping of the *Cf-16* gene

**FIGURE 3:**
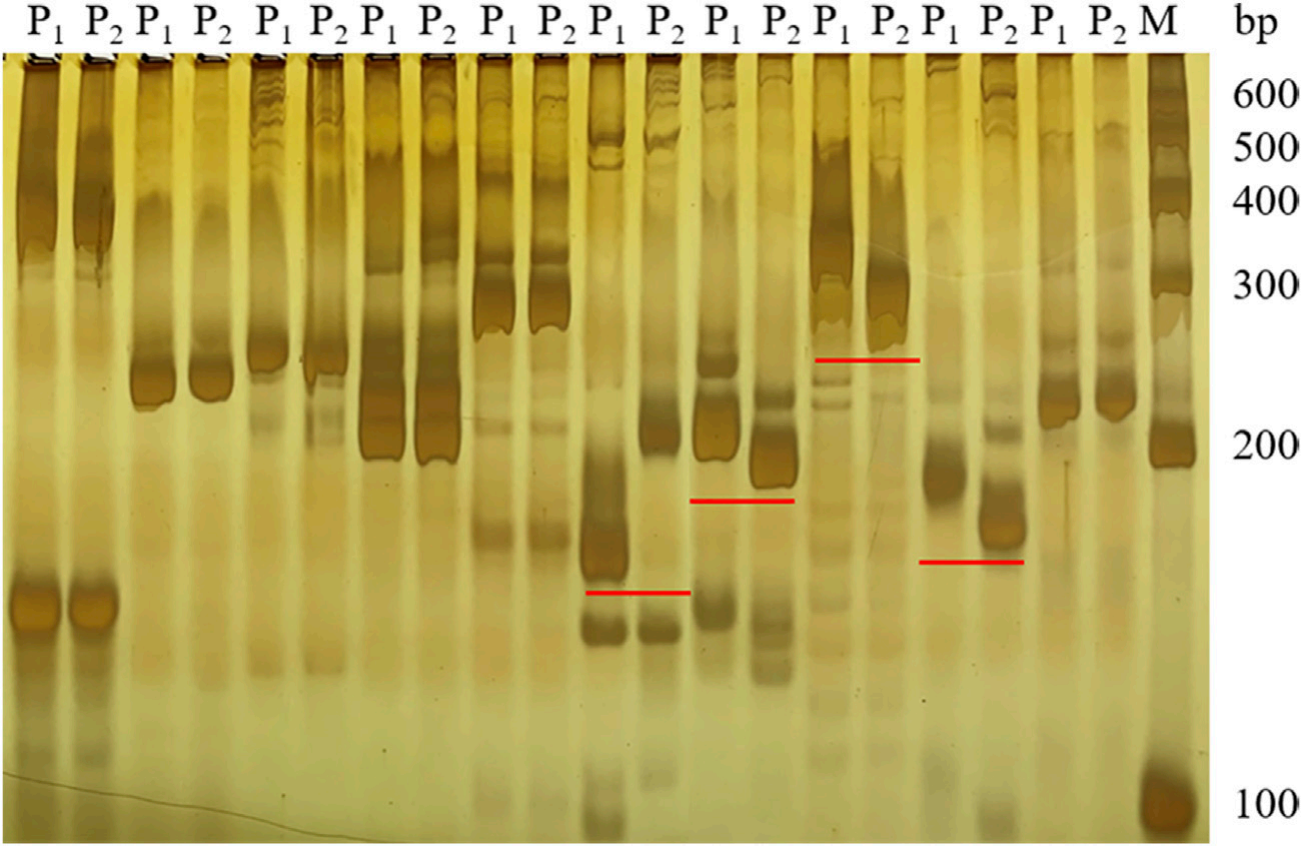
Screening of some SSR polymorphic markers.

**FIGURE 4:**
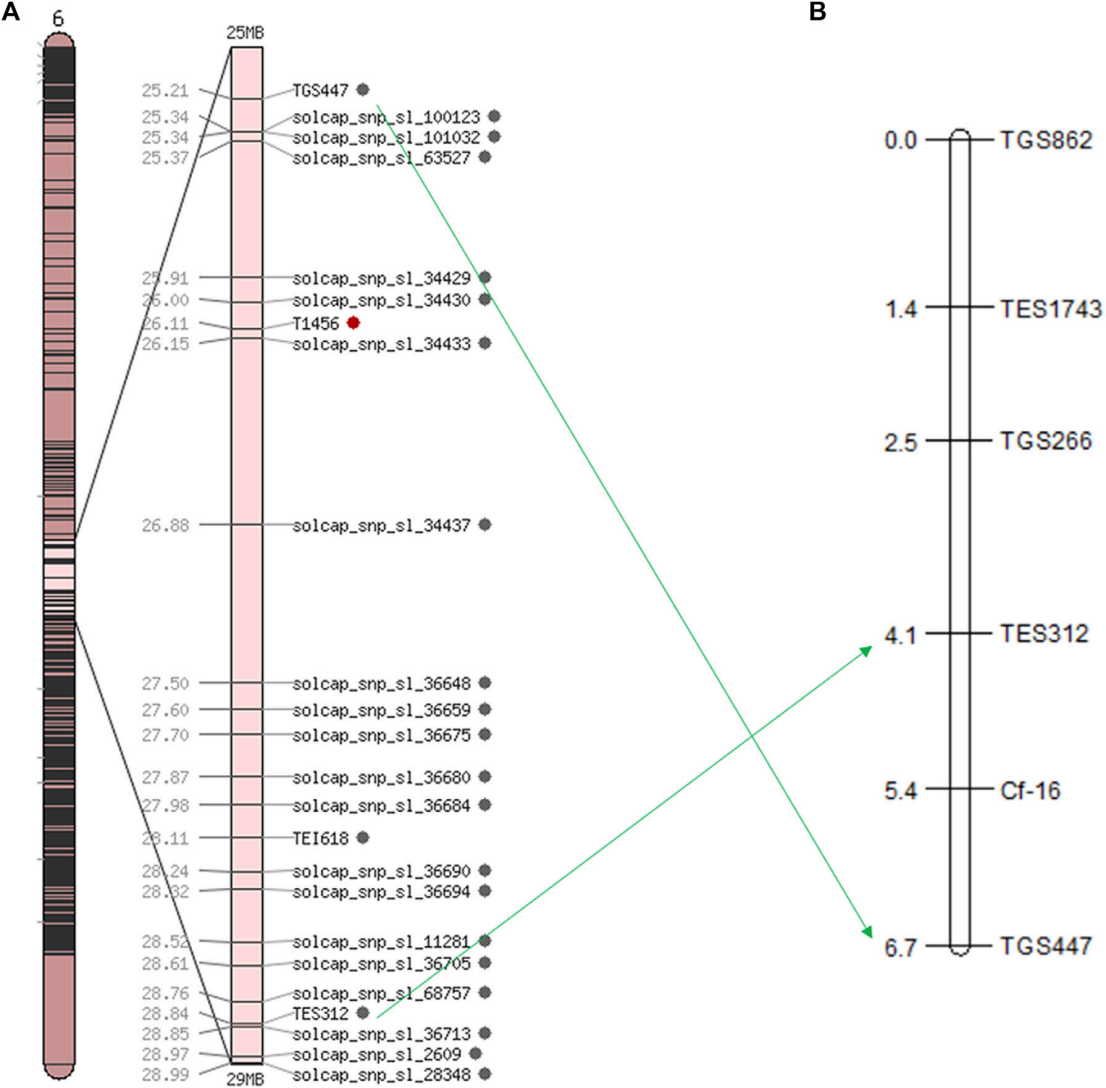
Linkage and chromosomal mapping of the leaf mold resistance gene *Cf-16* in tomato. **(A)** Genetic map of chromosome 6 in tomato; **(B)** SSR linkage of the *Cf-16* gene.

Based on the SNP index results, 58 SSR markers were used for polymorphic screening of the two parents and F_1_ generation to further map the *Cf-16* gene (Figure 3). Subsequently, SSR marker analysis was performed on 303 F_2_ generations. Linkage mapping was performed using JoinMap 4.0 software based on the phenotypes and marker types of the F_2_ generation (Figure 4). The results showed that the most closely linked markers to the *Cf-16* gene were TGS447 and TES312, both with a genetic distance of 1.3 cM. The positions of these two markers on chromosome 6 were 25.21 Mb and 28.84 Mb, respectively, and the physical distance of this candidate interval was 3.63 Mb.

### Analysis of candidate genes

Based on the candidate interval of the SNP index and SSR marker analysis, we found that 71 genes were distributed in the DNA fragment associated to the resistance gene *Cf-16*. Meanwhile, the number of nonsynonymous SNPs between the two parents in this region was 139. The 71 genes in the candidate interval were subjected to further functional annotation and structural analysis. Based on the typical structural features of the cloned tomato leaf mold resistance *Cf* gene encoding LRR-TM, 2 candidate genes were screened out, XM_004240667.3 and XM_010323727.1.

## Discussion

In recent years, tomato leaf mold has become probably the disease that damage tomato production the most. An effective way to control the disease is to breed resistance to leaf mold. However, some tomato leaf mold resistance (*Cf* series) genes used available hybrids using currently in farms have gradually lost resistance, due to the continuous and rapid differentiation of *C. fulvum* physiological races. It was found that the application of the *Cf-4* gene in production was overcome by *C. fulvum* physiological races such as 1.2.4 and 1.2.3.4. Subsequently, the application of *Cf-5* and *Cf-9*, which are commonly used in breeding, was gradually limited by the appearance and identification of physiological races 1.2.3.4.5, 1.2.3.4.9, 2.5, and 2.4.5 (Li et al., 2015; Zhao et al., 2016). It is crucial to understand the differentiation of *C. fulvum* physiological races and to characterize the resistance range of new *Cf* genes before undertaking breeding efforts for disease resistance. The *Cf-16* gene derived from the resistant material “Ontario7816” has shown effective resistance in field trials for many years. This study shows that “Ontario7816” has highly resistant to all the identified physiological races of *C. fulvum*. This result also indicates that this gene is a valuable resource against *C. fulvum* breeding. Therefore, mapping, cloning, and characterization of this gene may speed up its use in marker-assisted selection for resistance breeding and may have critical implications for our understanding of the resistance mechanism against tomato leaf mold disease.

Previous studies have shown that most *Cf* genes are inherited dominant single genes. In this study, the genetic analysis indicated that the segregation ratios between resistant and susceptible plants in both the F_2_ and BC_1_P_2_ populations were in accordance with Mendel’s law of segregation. The present results clarify that the *Cf-16* gene confers resistance via a single dominant gene. At the same time, it also paves the way for the next localization of *Cf-16*.

In this study, we mapped the *Cf-16* gene for the first time into a 2.6 cM region on tomato chromosome 6 between two markers, TGS447 and TES312, using BSA combined with parental resequencing and SSR molecular markers. By functional annotation and structural analysis of the genes in the candidate region, we ultimately predicted two candidate genes, XM_004240667.3 (LRR receptor-like serine-/threonine-protein kinase At1g51880) and XM_010323727.1 (LRR receptor-like serine-/threonine-protein kinase RFK1 isoform X1. LRR receptor-like serine-/threonine-protein kinase), the largest class of receptor-like protein kinase (RLK), has an LRR structure and transmembrane region composed of conserved amino acid sequences (Man et al., 2020). The protein structures encoded by the cloned *Cf* genes *Cf-2*, *Cf-4*, *Cf-5*, and *Cf-9* all have the typical extracellular LRR domain, transmembrane domain and a short cytoplasmic domain, and the different numbers of LRRs are responsible for different *Cf* genes recognizing different pathogen physiological races (Kruijt et al., 2005). The two genes that were ultimately predicted in this study may have similar structures to *Cf* genes that have been cloned, so they are identified as candidate genes for *Cf-16*. However, it is essential to conduct expression pattern analysis and further functional verification of these two candidate genes.

## Conclusion

In this study, we mapped the *Cf-16* gene for the first time into a 2.6 cM region on tomato chromosome 6 between two markers, TGS447 and TES312, using F_2_ bulked segregant analysis combined with genome resequencing and SSR molecular markers. Furthermore, we screened 2 possible candidate genes, XM_004240667.3 and XM_010323727.1, with LRR-TM structures similar to those of the cloned *Cf* genes according to their annotation information. The location and candidate gene screening of *Cf-16* could lay a robust foundation for later cloning of the *Cf-16* gene and applications in MAS selection programs.

## Data Availability

The datasets presented in this study can be found in online repositories. The names of the repository/repositories and accession number(s) can be found below: https://www.ncbi.nlm.nih.gov/sra PRJNA937877.
