# Supplementary material for: Mapping of the Cladosporium fulvum resistance gene Cf-16, a major gene involved in leaf mold disease in tomato
Source: Front Genet. 2023 Jul 27;14:1219898. doi: 10.3389/fgene.2023.1219898 (PMC10415096; doi:10.3389/fgene.2023.1219898)
Supplement: Supplementary file 1 [file Table1.docx]

**Table S1** Primers used for SSR molecular marker analysis

| SSR Primer | primer sequence-F | primer sequence-R | Chromosome Locus |
| --- | --- | --- | --- |
| TGS2236 | TGGTGGCCTGGTTTAGACTC | GCAAAACTGAACCAAAATGC | 6 |
| TES628 | GTCTCCTCGTTTATCCACGCT | TTCTCCACTTATGTGATTATACTGGG | 6 |
| TGS149 | GTTTCGTCAGTTGTTAAAAGTTGAAA | TGAAGCATTGGCTCAAAGAA | 6 |
| TES428 | GAGGGGGATGAAGTAGAGGC | TCCGACAGTGCAAAGTTCAG | 6 |
| TGS2204 | GCTTGACTTTTGCAGCCAACA | AAATAGCAAACAACTTACTCGAAAA | 6 |
| TES111 | ATCTCCTTGGCCTCCTGTTT | GTCATGGCCACATGAATACG | 6 |
| TES1873 | GTGTTCAAATTTGGTTTGGGC | AAAACCGCCAGGATATAGGC | 6 |
| TGS230 | GAAGCTGATGAACCCAGCAAT | CACATGTTTTGCATTTTTGTTG | 6 |
| TGS919 | TACTTATGTTCAAGGGGCCG | GGCAATTAGTGCATTCCGAT | 6 |
| TGS3509 | CAAAGTTCATTTGGGGGATG | GAGCATCATCAAATGCCTCA | 6 |
| TGS2108 | GTGTGTGTGGGCTGCTTACTC | TTGGGCAATGAAGAAGGAAG | 6 |
| SSR48 | ATCTCCTTGGCCTCCTGTTT | GTCATGGCCACATGAATACG | 6 |
| SSR47 | TCCTCAAGAAATGAAGCTCTGA | CCTTGGAGATAACAACCACAA | 6 |
| TGS2102 | TGATCATTGAGTTTTCTCCCTTC | GGGAATGTCAAGAGTGTTGGA | 6 |
| TES924 | TTGCAGATTGAGTCACGACA | GGCCCATCATTGTTTTCAAG | 6 |
| TGS917 | TGATGAAGCCCCTCAAGTTT | GTTGCGGTTCATGGGTAGAT | 6 |
| TGS871 | GTTCCTTGAACCAAACAAGCTC | TAGAATCCTTGTTTTGGCGG | 6 |
| TGS2480 | GACCTGCCTCATTTCCTTTCA | ATCGATGAAAACCATTCGGA | 6 |
| SSR128 | GGTCCAGTTCAATCAACCGA | TGAAGTCGTCTCATGGTTCG | 6 |
| TGS330 | GCAATGCGATAGTCTTCATGTCA | AAGTTTGTATTCGATTCACCCA | 6 |
| TGS447 | GATCCAAGGTTGGTTGCTTTG | CTTGGATGAGAAACCCCTGA | 6 |
| TES312 | GCTCTTCCCCAACCACCAATA | AAAGCCCTATTGGCCCTAGA | 6 |
| TGS1863 | GATGCTCAATAGCACAAGCCA | TTTTGTCCCTTTTTAGTGCGA | 6 |
| TES1743 | GAGTGTCTCGATCTCGCACCT | CCATGTGTCCAACCTTTTCC | 6 |
| TGS2216 | GAGGCTTCTAGCTCTGCCCTC | TTGAATTGGTCTGGGTCTCC | 6 |
| TGS3083 | GTCTCCAACGAAAAGAGGAGAA | TGGATGAGCTTGACATTCCA | 6 |
| TGS266 | GTGTAAGCAACCGCCATGTTA | TTTCGATTTAGGTGAACACGA | 6 |
| TGS862 | GTATGCAGTGGCGAAGTCAAG | CATATAGTCAGGGGCGGAGA | 6 |
| TGS2907 | TTCACCTCGGTTCAAACTCA | GCAAAGCACAACAAGACCAA | 6 |
| TES157 | CAGAAAACTCCAGTTCCCCA | GGGGTATTCCTAACAATAATCTGG | 6 |
| TES502 | AGGTTGAAAAGCAGCAAGGA | GTGCAACGTCGAAAGTGAAG | 6 |
| TES1094 | GCAACGTCATCTTCTCTCTTCCC | ATGCCAAGAAAATGGTGGAA | 6 |
| TGS3054 | GCACACTGACTCCGATTTCGTT | ACTTCCTTCCTATTGACCCAA | 6 |
| TES449 | GTCTCATTTGCTTAATTTCTTCTCC | CATCCCTCATTGCATCACTC | 6 |
| TES1190 | GACATCCAAACATGCTGGACA | AAGGAAATTGCACCGTCAAC | 6 |
| TES292 | GTCTGCACCAAAGAATCAATCA | AAGCTCTTTTGTGGGCTGAG | 6 |
| TES1179 | GCCAATCGCTAAATCCGCTTA | CACCAAGCCTCCTAATGCTG | 6 |
| TGS760 | GAAGAGAACTGAGCAAGGGAAGA | TTTTCAGTCATCTCTCCCGC | 6 |
| TES550 | GGAAGAACAAACCCCCATTT | AGAGGTTTTTCCATCCCCAAT | 6 |
| TES752 | GAATGACTCGGTGCATGTTG | ATTTACGCGCATTTGAAACC | 6 |
| TES211 | GTTTGCTTCAATTGTATATGTATAGCG | AGTTGACTCAGTGCCCGACT | 6 |
| TES298 | GTTTTCAAGCCAATGGTCGT | TGCGGTGGATATGAATTTGA | 6 |
| TGS3469 | GAACATTTCTGAAACGGTGGG | CCAAAATTCAGCCCTTTGAG | 6 |
| TES94 | GATGTGTGAGGCGTTGTTTGA | TTTTGCTATCATTCACATATTTCTTT | 6 |
| TGS1094 | GCTGTAAGCGAATCAAGTGC | TGCCATTGAACTCTCTGTGC | 6 |
| TES870 | CGTACGACGCTGTATCATGG | GTTTTGCCTCAAGAAGGTGC | 6 |
| TES702 | GATCCACCACCTCTCTCTCG | AATTCGAGTACGGCGAAGAA | 6 |
| TES1469 | GCTCTGCGTGGACTTTATCC | AAATGGGAGTCCCGTCTTCT | 6 |
| TES335 | TAATTGGGGTGCAAGAAAGG | GCCGTTTTACAATTAGGGCA | 6 |
| TGS2253 | GCAAAGCTTTAAGTAGTGGCG | TTCTTCACATTGTTTTTGGTGAA | 6 |
| TGS2372 | GTTTGCTTTTCGGATTTGGAC | CGTAAACAAAGGGGAAGCAC | 6 |
| TGS467 | GATATGAATCGGGTTGGCTTG | CGTCCATCTTGTTGGCTCTT | 6 |
| SSR128 | GGTCCAGTTCAATCAACCGA | TGAAGTCGTCTCATGGTTCG | 6 |
| TES355 | GAGGCAGATATCAGCGATGG | CATGAACTCTTGGCGGATTT | 6 |
| TGS892 | GGTCCGTACCTCTTTTTCCC | AGGCATAGCGGCTGAGATAGA | 6 |
| TGS228 | GTCCTTTCTTGTCAAGCAGCC | TGGACCACACAAAAGTTCCA | 6 |
| TES1041 | GCCTTCTCCCACTGAACCCTA | CTTCACGAACCTCTTCGGAC | 6 |
| TES1805 | GATGCAAATTCAGGGGATTCA | CAAATGAAATCAAAATGCTTCC | 6 |
