# Supplementary figures and images for: Mapping of the Cladosporium fulvum resistance gene Cf-16, a major gene involved in leaf mold disease in tomato
Source: Front Genet. 2023 Jul 27;14:1219898. doi: 10.3389/fgene.2023.1219898 (PMC10415096; doi:10.3389/fgene.2023.1219898)

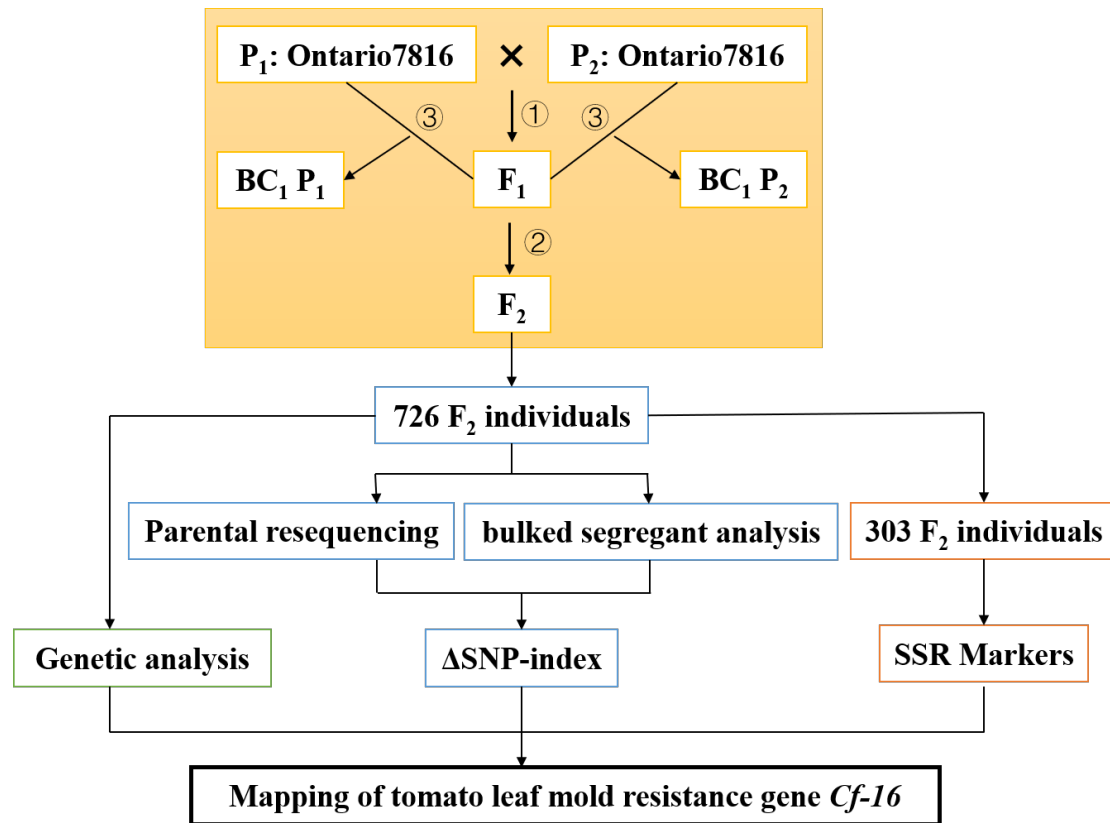

Figure S1: Flow chart of mapping the *Cf-16* gene in 'Ontario7816'.

Supplement: Supplementary file 2 [file Image1.pdf]
